# Draft Genome Sequence of *Marinobacter* sp. Strain C7 Isolated from Seawater in Con Bung Coast, Vietnam

**DOI:** 10.1128/mra.00404-22

**Published:** 2022-06-08

**Authors:** Ngoc-Lan Nguyen, Van Dung Vu, Van Tung Nguyen, Thi Kim Lien Nguyen, Huy Hoang Nguyen

**Affiliations:** a Institute of Genome Research, Vietnam Academy of Science and Technology, Cau Giay, Hanoi, Vietnam; b Graduate of Science and Technology, Vietnam Academy of Science and Technology, Cau Giay, Hanoi, Vietnam; c Institute of Chemistry and Materials, Academy of Military Science and Technology, Cau Giay, Hanoi, Vietnam; University of Southern California

## Abstract

*Marinobacter* sp. strain C7 was isolated from seawater collected on the Con Bung coast, Vietnam. Here, we report a draft genome sequence of strain C7 consisting of 4,057,300 bp with 59.2% GC content and 109 contigs. The genome sequence of strain C7 provides an overview of its halophilic properties.

## ANNOUNCEMENT

*Marinobacter* sp. strain C7 was isolated from seawater collected on the Con Bung coast, Ben Tre province, Vietnam (9°50'00.2″N, 106°39′34.9″E), using marine agar medium (Gellix, South Korea) supplemented with 5% (wt/vol) NaCl and incubated at 30°C in April 2017 (1). The strain was maintained in marine agar and marine broth. The 16S rRNA analysis classified strain C7 (GenBank accession number MK850328) as a member of the genus *Marinobacter* (1). The closest relative, Marinobacter pelagius strain HS225^T^ (GenBank accession number DQ458821), shared 99.09% 16S rRNA gene sequence identity with strain C7 (1). Strain C7 was able to grow at 1 to 17% (wt/vol) NaCl (optimal, 4%); therefore, strain C7 was characterized as a moderately halophilic bacterium (1).

A single colony of strain C7 was streaked onto marine agar supplemented with 2% (wt/vol) NaCl and incubated at 30°C for 24 h. An inoculation loop of bacterial cells was collected for genomic DNA extraction using the Qiagen DNeasy Blood & Tissue kit (Qiagen, USA) in accordance with the manufacturer’s instructions. Quantities of extracted DNA were measured with a NanoDrop 1000 spectrophotometer (Thermo Scientific, USA). The library was prepared using the NEBNext Ultra DNA library prep kit (Illumina) following the manufacturer’s protocol. Sequencing was carried out using a 2 × 150 paired-end (PE) configuration on the HiSeq instrument at Genewiz Biological Technology Co., Ltd. (Suzhou, China). Sequencing released 17,568,606 pairs of reads. The pair-ended low-quality reads were filtered out using Trimmomatic v0.39 (2). The assembly of the contigs was carried out using SPAdes v3.15.3 (3), and contigs of less than 500 bp were excluded. The contaminated contigs of the initial assembly were detected by the NCBI contamination screen and removed. The size and quality of the final assembly were evaluated using QUAST v5.0.2 (4). Gene prediction and annotation were performed using the NCBI Prokaryotic Genome Annotation Pipeline (PGAP) v6.0 (5). Default parameters were used for all software unless otherwise specified.

The final draft genome of strain C7 consists of 4,057,300 bp with a mean coverage of 318×, spanning 109 contigs with 59.2% GC content. The *N*_50_ value is 2,152,396 bases, and the *L*_50_ value is 1 contig. The genome includes 3,797 genes with 3,690 protein-coding genes, 5 rRNA, 46 tRNA, and 1 transfer-messenger RNA (tmRNA).

**FIG 1:**
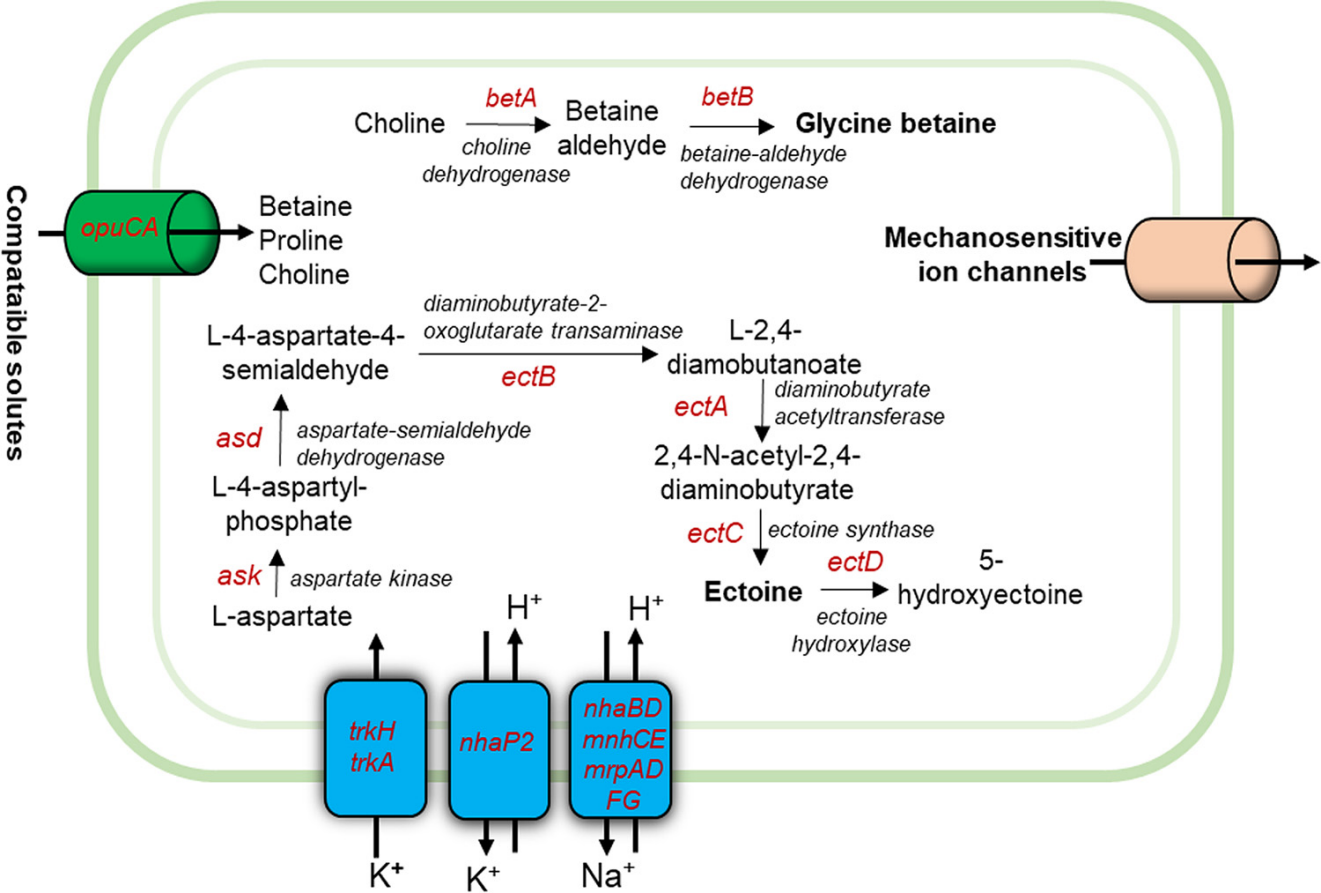
Illustration of potential salt tolerance mechanism of *Marinobacter* sp. strain C7. *OpuCA*, betaine/proline/choline family ABC transporter ATP-binding protein; *trkAH*, potassium uptake system proteins; *nhaP2m*: K^+^/H^+^ antiporter; *nhaBD*, *mnhCE*, and *mrpADFG*, Na^+^/H^+^ antiporters.

The putative genes responsible for modulation of cytoplasmic solute levels and exclusion of salt from the cytoplasm were identified in strain C7 (Fig. 1). Therefore, we propose that strain C7 uses both salt-in and salt-out strategies to sustain growth at high salinity.

### Data availability.

The genome sequencing project has been deposited in GenBank under the BioProject accession number PRJNA805009. The draft genome assembly of *Marinobacter* sp. strain C7 has been deposited in GenBank under the accession number JAKQZH000000000. The version described in this paper is the first version, JAKQZH010000000. The raw sequencing reads are available under SRA accession number SRR18652104.
